# Adherence thresholds of tenofovir diphosphate in dried blood spots for HIV treatment and pre-exposure prophylaxis: A scoping review

**DOI:** 10.4102/sajhivmed.v27i1.1829

**Published:** 2026-09-02

**Authors:** Mohammed K. Alghamdi, Mohammed W. Ali, Anel Schoonees, Tamara Kredo, Eric Decloedt, Roland van Rensburg

**Affiliations:** 1 Division of Clinical Pharmacology, Faculty of Medicine and Health Sciences, Stellenbosch University, Cape Town, South Africa; 2 Department of Clinical Pharmacology, Faculty of Medicine, King Abdulaziz University, Jeddah, Saudi Arabia; 3 Division of Epidemiology and Biostatistics, Faculty of Medicine and Health Sciences, Stellenbosch University, Cape Town, South Africa; 4 Health Systems Research Unit, South African Medical Research Council, Cape Town, South Africa

**Keywords:** tenofovir diphosphate, dried blood spots, adherence, pre-exposure prophylaxis, antiretroviral therapy, threshold, scoping review, analytical variability

## Abstract

**Background:**

Tenofovir (TFV) is a key antiretroviral used in HIV pre-exposure prophylaxis (PrEP) and treatment, with efficacy contingent on adherence. Quantifying TFV concentrations in dried blood spots (DBS) as tenofovir diphosphate (TFV-DP) is a robust method for determining long-term adherence. However, standardised TFV-DP adherence thresholds in DBS have not been established.

**Objectives:**

To map the existing literature on TFV-DP concentration thresholds in DBS for assessing adherence to TFV-based HIV prevention and treatment, and to identify evidence gaps.

**Method:**

We conducted a scoping review in accordance with Preferred Reporting Items for Systematic Reviews and Meta-Analyses extension for Scoping Reviews guidelines. Eight electronic databases were searched for studies reporting TFV-DP concentrations in DBS in relation to adherence thresholds or clinical outcomes for TFV-based PrEP or antiretroviral therapy (ART). Pharmacokinetic simulation or modelling studies used as reference benchmarks were included. Data were screened and extracted by two independent reviewers. We conducted descriptive analysis, tabulating findings.

**Results:**

Twenty-two studies were included. TFV-DP adherence thresholds for PrEP and treatment varied widely, from ≥ 650 fmol/punch to ≥ 1850 fmol/punch, reflecting differences in study design, populations and ART formulations. Reference studies defined adherence threshold as ≥ 700 fmol/punch for four doses/week. PrEP studies reported TFV-DP concentrations of 993–1173 fmol/punch for 4–7 doses/week, indicating that the reference threshold was conservative. In treatment studies, concentrations ≥ 1250 fmol/punch were associated with virological suppression, whereas concentrations < 800 fmol/punch were linked to an increased risk of viraemia.

**Conclusion:**

This review of TFV-DP in DBS for HIV PrEP and treatment supports purpose- and population-specific thresholds and highlights the need for further threshold validation to optimise PrEP and treatment strategies.

**What this study adds:** This scoping review maps TFV-DP adherence thresholds in DBS for HIV PrEP and treatment, demonstrating substantial variability across reported thresholds. It highlights the need for purpose- and population-specific thresholds, and identifies key methodological and biological factors influencing interpretation.

## Background

Improving pre-exposure prophylaxis (PrEP) and HIV treatment outcomes requires adequate adherence to antiretroviral therapy (ART). Traditional assessment methods of ART adherence such as self-reports, pill counts, and pharmacy refill records are widely used, but have important limitations. For example, self-reports are prone to recall bias and social desirability bias, while pill counts and pharmacy refill records may not reflect actual ingestion of ART.^1^ To address these limitations, quantification of antiretroviral concentrations in plasma was developed.^2^

However, the utility of plasma antiretroviral concentrations to assess adherence beyond the preceding dose is limited because of the short plasma half-lives of most drugs.^3^ Recent adherence behaviour, particularly where patients display ‘white-coat adherence’ by improving ART intake just before their clinic visit, may result in apparent adequate plasma concentrations despite overall poor adherence.^4^ Tenofovir diphosphate (TFV-DP), the intracellular active metabolite of the common first-line ART, tenofovir, accumulates in red blood cells with a half-life of approximately 17 days when tenofovir disoproxil fumarate is administered,^5^ and has therefore gained interest as a measure of longer-term adherence. Subsequently, intracellular TFV-DP concentrations allow for the estimation of average adherence over the preceding 6 to 8 weeks at steady state, thus providing an objective measure of cumulative adherence.^5,6^ TFV-DP can be measured reliably in dried blood spots (DBS), where the drying process preserves the analyte and allows for sampling at clinic sites that are removed from the processing laboratories.^5,7^

Quantification of TFV-DP concentrations is currently being used as a research tool and is not yet readily available to inform individual patient care.^8,9^ Reasons for the current lack of uptake into clinical practice include the need for access to specialised analytical pharmacology infrastructure and expertise, cost of the assay, and the interpretation of TFV-DP concentrations. There is currently no consensus on the cut-off thresholds of TFV-DP concentrations to distinguish adequate from inadequate adherence to achieve virological suppression for HIV treatment, or prevention of HIV transmission for PrEP. The lack of consensus is likely a result of the variability of TFV-DP concentrations, which may be explained by variations in populations, study designs, and laboratory methodologies.^8,10,11,12^ We therefore performed a scoping review to map the extent, nature, and range of evidence on TFV-DP threshold values, given the diverse methodologies and varying definitions used in the literature. Scoping reviews enable the identification of knowledge gaps, support harmonisation efforts, and inform future research and policy directions.

The aim of this scoping review was therefore to map the current evidence on TFV-DP adherence thresholds in DBS, to describe variations across populations and study methods, and to identify key gaps for further research.

## Research methods and design

We conducted this scoping review in accordance with the Preferred Reporting Items for Systematic Reviews and Meta-Analyses extension for Scoping Reviews (PRISMA-ScR) guidelines.^13^ The protocol was prospectively registered on the Open Science Framework (https://osf.io/72n9b). Reporting was done according to the PRISMA-ScR checklist (Online Appendix 1 Figure 1-A1).

### Study objectives

The scoping review sought to answer the research question: ‘What are the published thresholds for TFV-DP in DBS for evaluating adherence to TFV-based ART for HIV prevention and treatment?’

The objectives of the review were:

- To map the existing literature on TFV-DP concentration thresholds in DBS for assessing adherence to TFV-based HIV prevention and treatment strategies.
- To identify gaps in the current evidence related to TFV-DP concentration thresholds for adherence assessment.

### Eligibility criteria

Eligibility criteria for this review were defined using the Population, Concept, and Context framework:

#### Population

The review included human participants of any age, sex, HIV status, or geographic location who were treated with TFV-based ART for PrEP and treatment. Animal studies and laboratory-based studies without human participants were excluded.

#### Concept

The review included studies measuring TFV-DP concentrations in DBS as a marker of adherence, regardless of study design, or methodology. Eligible studies were those that reported adherence thresholds (number of doses/week) or analysed clinical outcomes in relation to TFV-DP concentrations. Optimal adherence was defined as participants taking all doses prescribed. Adequate adherence was defined as taking the number of prescribed doses needed to achieve clinical protection, such as a reduced risk of HIV acquisition in PrEP studies or virological suppression in treatment studies.

#### Context

Studies were considered regardless of the healthcare or research setting, including clinical trials, community-based studies, or laboratory investigations. The review focused on studies using quantitative methods, such as pharmacokinetic (PK) analyses, cross-sectional designs, or cohort studies. Narrative reviews, commentaries, and other non-primary literature were excluded.

### Evidence sources

We included interventional studies, observational studies, and systematic reviews examining TFV-DP concentrations in DBS linked to adherence thresholds for HIV treatment and PrEP. Eligible studies encompassed randomised controlled trials, pre–post studies, and observational research (cohort, case-control, cross-sectional studies, and case series/reports). Systematic reviews needed clear objectives, inclusion criteria, searches of multiple databases, and formal data extraction and bias assessment. The search strategy prioritised published studies, followed by a structured search for unpublished data, including conference abstracts and registered study protocols. Where identified primary studies were also included in systematic reviews, we used the primary studies and checked reference lists of the systematic reviews for completeness. Published protocols of registered and/or ongoing studies were included if they met the eligibility criteria.

### Search strategy

A comprehensive search was performed across multiple electronic databases up to March 2024. An updated search was subsequently performed, covering the period from April 2024 to December 2025 to identify newly published evidence relevant to TFV-DP adherence thresholds. The databases searched involved MEDLINE (PubMed), The Cochrane Library (including CDSR and CENTRAL), Web of Science Core Collection, Africa-Wide Information (EBSCOhost), Epistemonikos. In addition, trial and systematic review registries were searched, including PROSPERO, ClinicalTrials.gov, and the WHO International Clinical Trials Registry Platform. Search terms included ‘tenofovir diphosphate’, ‘dried blood spot’, and ‘adherence’. The search strategy was tailored to each database to optimise retrieval of relevant studies. A search history document was maintained for transparency, detailing search queries, filters, and records retrieved. The full search strategy can be found in Online Appendix 1 Figure 2 A-1.

In addition to electronic databases, we also conducted a manual search of reference lists from eligible records, and reviewed grey literature, including conference proceedings, abstracts, and presentations from directed professional meetings (the Conference on Retroviruses and Opportunistic Infections, and the HIV Treatment and Prevention Adherence Conference) from 2022 to 2024, as these were the most recent years at the time of our search and aligned with the study’s aim to capture the current evidence on TFV-DP adherence thresholds. All retrieved studies were imported into Rayyan software (https://www.rayyan.ai) for duplicate removal and screening.

### Data management, extraction, and analysis

Following duplicate removal, two reviewers independently screened titles, abstracts, and full texts. Disagreements were resolved through discussion, or by consultation with a third reviewer. Data were extracted into predefined headings, capturing study details, participant characteristics, adherence assessment methods, TFV-DP thresholds in DBS, and clinical outcomes. The extraction tool was refined as needed to account for variation across studies. Formal critical appraisal of included studies was not performed, consistent with the scoping review objective of mapping the available evidence rather than assessing intervention effectiveness. TFV-DP thresholds were interpreted according to the primary objective of the included study, including PK-derived adherence benchmarks from the number of doses per week, and thresholds associated with clinical outcomes such as virological suppression, future viraemia, or new HIV acquisitions. A descriptive synthesis was conducted, and key findings were summarised in tables to highlight threshold ranges, methodological differences, and evidence gaps. In addition, a post hoc graphical summary was developed to visually compare the range and distribution of reported TFV-DP threshold values across different study categories.

### Ethical considerations

This article does not contain any studies involving human participants performed by any of the authors and followed all ethical standards for research without direct contact with human or animal subjects.

## Results

### Study selection

Our comprehensive database search identified 733 records (Figure 1). An additional eight records were retrieved through manual searches of reference lists and grey literature, leading to a total of 741 records. After removing duplicates, 439 records remained for title and abstract screening. Of these, 235 records were excluded for not reporting TFV-DP concentrations in DBS.

**FIGURE 1:**
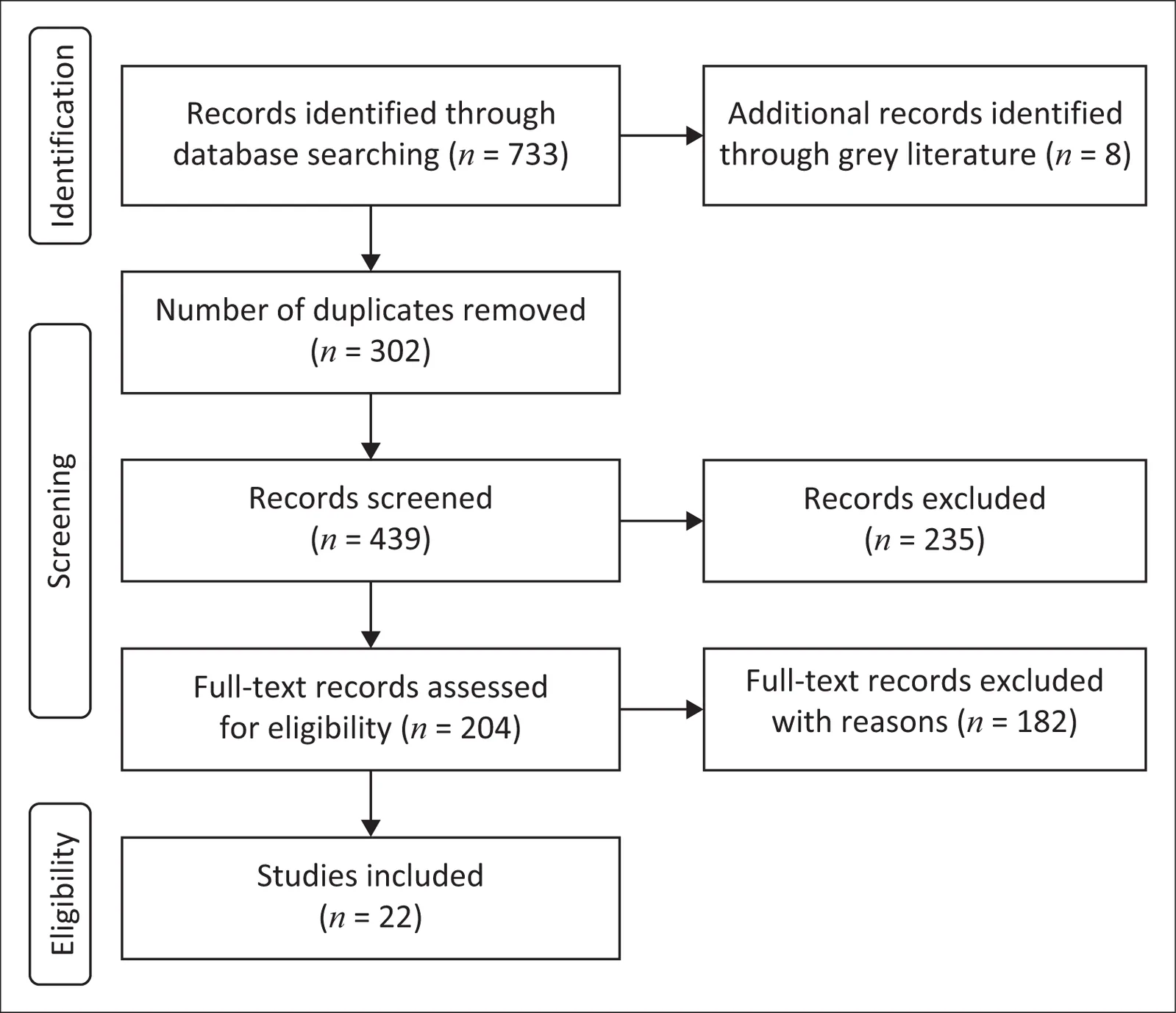
Preferred Reporting Items for Systematic Reviews and Meta-Analyses flow diagram.

Subsequently, 204 full-text records were assessed for eligibility. Of these, 182 records were excluded with reasons (Online Appendix 1 Table 1 A-1), and 22 studies were included in the final synthesis. The updated search conducted from April 2024 to December 2025 was incorporated into the final PRISMA flow diagram.

The included studies were categorised into three clusters based on their methods and populations: reference studies, PrEP studies, and treatment studies. Reference studies refer to studies that either used simulations, PK modelling, or directly observed dosing to establish the association between the number of doses taken and TFV-DP concentrations in DBS under controlled conditions. Reference studies are frequently cited and used as reference by PrEP and treatment studies to interpret findings. These studies typically defined the TFV-DP concentrations expected with specific dosing frequencies (e.g. 2, 4, or 7 doses per week) and provided a framework for interpreting adherence in applied settings. PrEP studies investigated adherence to ART and its efficacy in preventing HIV in high-risk populations without HIV. Treatment studies examined adherence to ART in persons living with HIV (PLWH) with the clinical outcome of virological suppression. In this review, TFV-DP thresholds were interpreted according to the primary objective of the included study. Reference studies primarily generated PK-derived adherence benchmarks by relating TFV-DP concentrations to estimated dosing frequency under controlled conditions, whereas PrEP and treatment studies evaluated TFV-DP concentrations in relation to HIV prevention outcomes and treatment studies evaluated TFV-DP concentrations in relation to virological outcomes. Although related, these thresholds are not directly interchangeable, as adherence-associated TFV-DP concentrations may differ from thresholds associated with clinical outcomes, depending on ART regimen, population characteristics, and study methodology.

### Demographic and clinical characteristics of participants in the included studies

#### Reference studies

Ten reference studies were identified (Table 1) which included 2037 participants, mostly from high-income countries. Sample sizes ranged from 12 to 1603 participants. These studies included people without HIV aged 18 to 49 years, and peripartum women with and without HIV. Most reference studies were performed under controlled laboratory or clinical conditions in the United States,^5,10,11,14,15,16^ with some representation from sub-Saharan Africa and Asia (Thailand), extending the evaluation of TFV-DP thresholds to more diverse and resource-limited countries.^6,8,17,18^

**TABLE 1:** Demographic and clinical characteristics of all included studies.

Study author	Location	Study design	Participants		Age			
			*n*	% female	Mean	Median	Range	s.d.
**Reference studies**								
Castillo-Mancilla et al.^5^	United States	PK study with simulation	17	59	-	30	22–47	-
Anderson et al.^16^	United States	PK study with randomised crossover design	48	52	-	29	-	-
Grant et al.^14^	United States	Prospective cohort study	1603	0	18–25	-	-	-
Yager et al.^10^	United States	PK study with crossover design	37	46	-	29	18–41	-
Ibrahim et al.^11^	United States	PK study with simulation	48	52	-	29	21–49	-
Devanathan et al.^15^	United States	PK study with simulation	12	‡	‡	‡	‡	‡
Stranix-Chibanda et al.^8^	Sub-Saharan Africa†	Prospective cohort with simulation	40	100	-	20	19–22	-
Abuogi et al.^6^	Kenya	Nested prospective cohort study	150	100	-	28	25–32	-
Niu et al.^17^	Thailand	PK study with randomised parallel design	28	43	-	33	28–40	-
Mugwanya et al.^18^	Kenya	PK study with randomised parallel design	54	100	-	22	20–25	-
**PrEP studies**								
Hoenigl et al.^19^	United States	Prospective cohort study	122	0	37	-	-	10
Pyra et al.^20^	Kenya, Uganda	Prospective cohort study	103	34	31.7	-	-	9.2
Wahome et al.^21^	Kenya	Prospective cohort study	170	0	-	25	19–44	-
Landovitz et al.^22^	28 countries (global)	Pooled analysis of 72 PrEP studies	17 274	32	-	28	14–77	-
**Treatment studies**								
Castillo-Mancilla et al.^25^	United States	Cross-sectional study	35	100	-	42	-	-
Castillo-Mancilla et al.^9^	United States	Prospective cohort study	532	14	-	46	33–51	-
Castillo-Mancilla et al.^27^	United States	Prospective cohort study	521	14	-	46	36–52	-
Morrow et al.^26^	United States	Prospective cohort study	451	16	37	-	-	10
Robbins et al.^24^	United States	Prospective cohort study	61	69	24.2	-	-	2.7
Jennings et al.^23^	South Africa	Prospective cohort study	250	78	-	34	27–42	-
Odayar et al.^7^	South Africa	Nested case-control study	81	100	-	29	26–32	-
Van Heerden et al.^28^	South Africa	Post hoc analysis of two randomised controlled trials	294	57	-	36	31–44	-

Note: Please see the full reference list of Alghamdi MK, Ali MW, Schoonees A, Kredo T, Decloedt E, Van Rensburg R. Adherence thresholds of tenofovir diphosphate in dried blood spots for HIV treatment and pre-exposure prophylaxis: A scoping review. S Afr J HIV Med. 2026;27(1), a1829. https://doi.org/10.4102/sajhivmed.v27i1.1829

PK, pharmacokinetic; PrEP, Pre-exposure prophylaxis; s.d., standard deviation.

† , Sub-Saharan countries included Malawi, South Africa, Uganda, and Zimbabwe;

‡ , not reported.

#### PrEP studies

Four PrEP studies were included (Table 1). Three were prospective cohort studies with sample sizes ranging from 103 to 170 participants,^19,20,21^ conducted in the United States, Kenya, and Uganda. The fourth study was a large pooled analysis of 72 PrEP studies, including 17 274 participants across 28 countries, not including the three prospective cohort studies mentioned above.^22^ Participants were predominantly adults, with median ages ranging from the mid-20s to late-30s, and men (approximately 66% – 100% in earlier cohort studies and 63% in the large pooled analysis).

#### Treatment studies

Eight treatment studies were identified (Table 1) which included 2226 participants, with sample sizes between 36 and 532 participants.^7,9,23,24,25,26,27^ Most studies were conducted in the United States, with three studies conducted in South Africa. Ages ranged from 18 to 52 years and included a higher proportion of women, particularly in peripartum and postpartum studies. Overall, 741 participants were women, representing 33% of the total treatment cohort.

### Tenofovir diphosphate in dried blood spot thresholds

#### Reference studies

Reference studies used a range of primary study designs, including PK studies, randomised crossover trials, and prospective cohort studies to establish TFV-DP concentrations in DBS based on the number of doses. The data from these studies were used to determine definitions of adherence correlated to TFV-DP concentrations in DBS with different dosing regimens and were cited by subsequent studies in the PrEP and treatment categories (Table 2). The study populations included people without HIV, men who have sex with men (MSM), and peripartum women using PrEP or ART. The duration of the included studies ranged from 1 to 18 months. An adequate adherence threshold for PrEP to prevent HIV transmission was commonly defined as four doses per week, corresponding to a TFV-DP concentration of ≥ 700 fmol/punch in DBS. This threshold was not derived as a pooled average across studies but is based on the most commonly reported concentration from controlled PK and observational studies with directly observed dosing, including Castillo-Mancilla et al.,^5^ Anderson et al.,^16^ and Grant et al.,^14^ which estimated TFV-DP concentrations associated with specific dosing frequencies. However, it was not consistently observed across studies, and the TFV-DP concentrations associated with similar adherence levels often varied by population and study context. For example, in a PK study conducted in Asia, Niu et al.^17^ reported TFV-DP concentrations of approximately 779 fmol/punch associated with adherence equal to or exceeding four doses per week, which highlights variability around the commonly reported ≥ 700 fmol/punch benchmark. TFV-DP concentrations also varied by ART regimen, with lower concentrations observed with efavirenz-based regimens compared with integrase inhibitor-based regimens despite similar adherence and virological suppression (E. Decloedt, personal communication, 2025, unpublished data).

**TABLE 2:** Results from reference studies by region.

Study author	Participants	Intervention (daily)	Duration	Definition of adequate† or optimal‡ adherence	Cut-off value for TFV-DP in DBS
**United States**					
Castillo-Mancilla et al.^5^	People without HIV	TDF 300 mg or FTC 200 mg	1 month	Adequate adherence for PrEP: 4 doses/week	≥ 900 fmol/punch
Anderson et al.^16^	People without HIV	TDF 300 mg or FTC 200 mg	3 months	Adequate adherence for PrEP: 4 doses/week	≥ 700 fmol/punch
Grant et al.^14^	People without HIV	TDF 300 mg or FTC 200 mg	18 months	Adequate adherence for PrEP: ≥ 4 doses/week	≥ 700 fmol/punch
Yager et al.^10^	People without HIV	TAF (25 mg) or FTC (200 mg	9 months	Adequate adherence for PrEP: ≥ 4 doses/week	≥ 950 fmol/punch
Ibrahim et al.^11^	People without HIV	TDF 300 mg or FTC 200 mg	3 months	Adequate adherence for PrEP: 2–3 doses/week (≤ 110 kg) and ≥ 4 doses/week (> 110 kg)	≥ 700 fmol/punch
Devanathan et al.^15^	People without HIV	TDF 300 mg or FTC 200 mg	9 months	Adequate adherence for PrEP: ≥ 4 doses/week	≥ 1000 fmol/punch
**Africa**					
Stranix-Chibanda et al.^8^	Peripartum women without HIV	TDF 300 mg or FTC 200 mg	3 months	Optimal adherence for PrEP: 7 doses/week	≥ 650 fmol/punch in pregnancy ≥ 1050 fmol/punch postpartum
Abuogi et al.^6^	Peripartum women with HIV	TDF 300 mg-based ART	12 months	Optimal adherence for HIV treatment in peripartum women: 7 doses/week	≥ 650 fmol/punch in pregnancy ≥ 950 fmol/punch postpartum
Mugwanya et al.^18^	People without HIV	TDF 300 mg or FTC 200 mg	8 weeks	Adequate adherence for PrEP: > 4 doses/week	≥ 700 fmol/punch (70:30 extraction) ≥ 900 fmol/punch (50:50 extraction)
				Optimal adherence for PrEP: 7 doses/week	≥ 1250 fmol/punch (70:30 extraction) ≥ 1600 fmol/punch (50:50 extraction)
**Asia**					
Niu et al.^17^	People without HIV	TDF 300 mg/FTC 200 mg	10 weeks	Adequate adherence for PrEP: > 4 doses/week	779 fmol/punch
				Optimal adherence for PrEP: 7 doses/week	1375 fmol/punch

Note: Please see the full reference list of Alghamdi MK, Ali MW, Schoonees A, Kredo T, Decloedt E, Van Rensburg R. Adherence thresholds of tenofovir diphosphate in dried blood spots for HIV treatment and pre-exposure prophylaxis: A scoping review. S Afr J HIV Med. 2026;27(1), a1829. https://doi.org/10.4102/sajhivmed.v27i1.1829

ART, antiretroviral therapy; DBS, dried blood spots; FTC, Emtricitabine; PrEP, pre-exposure prophylaxis; TAF, tenofovir alafenamide; TDF, tenofovir disoproxil fumarate; TFV-DP, tenofovir diphosphate.

† , Taking the number of prescribed doses needed to achieve the reported TFV-DP concentration on DBS;

‡ , Participants taking all prescribed doses.

Methodological factors also influenced threshold interpretation. Mugwanya et al.^18^ showed that TFV-DP concentrations varied according to DBS extraction methodology, with lower concentrations observed using a 70:30 methanol–water extraction compared with a 50:50 extraction. In this study, adequate adherence (> 4 doses/week) corresponded to ≥ 700 fmol/punch (70:30) and ≥ 900 fmol/punch (50:50), while optimal adherence (7 doses/week) corresponded to ≥ 1250 fmol/punch (70:30) and ≥ 1600 fmol/punch (50:50).

Beyond methodological differences, several population-specific and physiological factors contributed to variability in TFV-DP thresholds across reference studies. For example, studies in peripartum women without HIV defined optimal PrEP adherence as seven doses per week, which corresponds to ≥ 650 fmol/punch during the second to third trimesters of pregnancy, and to ≥ 1050 fmol/punch in the postpartum period.^6,8^ Variability was observed in adequate adherence definitions based on body weight, pregnancy status, and TFV formulation. Ibrahim et al.^11^ introduced a model incorporating weight-based adherence benchmarks, showed that participants weighing ≤ 110 kg required only two to three doses per week to achieve TFV-DP concentrations similar to those weighing > 110 kg taking four doses per week.

#### PrEP studies

The smaller PrEP studies from the United States and Africa included MSM and serodiscordant couples,^19,20,21^ whereas the large global pooled analysis included diverse populations^22^ (Table 3). These studies quantified TFV-DP concentrations in DBS to assess adherence and correlated it with HIV prevention outcomes. While the reference studies established the relationship between dosing frequency and TFV-DP concentrations, PrEP studies reported large variability in observed TFV-DP concentrations, suggesting differences in adherence patterns across populations.

**TABLE 3:** Results of pre-exposure prophylaxis studies by region.

Study Author	Participants	Intervention (daily)	Duration (months)	Definition of adequate† or optimal‡ adherence			Observed value for TFV-DP in DBS				% Target attainment or outcome
				Adherence	fmol/punch	Doses/week	Median (fmol/punch)	IQR (fmol/punch)	Mean (fmol/punch)	s.d.	
**United States**											
Hoenigl et al.^19^	MSM	TDF 300 mg or FTC 200 mg	12	Adequate	719	≥ 4	993	0–1397	-	-	76% of participants had adequate adherence at 48 weeks post-trial
				Optimal	1246	7	1173	0–1533	-	-	35% of participants had optimal adherence at 48 weeks post-trial
**Africa**											
Pyra et al.^20^	Serodiscordant couples	TDF 300 mg or FTC 200 mg	± 11	Adequate	≥ 700	4	-	-	925	509	58% of DBS samples met the ≥ 4-dose adherence target
				Optimal	≥ 1050	6	-	-	994	517	41% of DBS samples met the ≥ 6-dose adherence target
Wahome et al.^21^	MSM	TDF 300 mg or FTC 200 mg	12	Adequate	≥ 700	4	§	§	§	§	Only 14.5% had TFV-DP ≥ 700 fmol/punch 57.9% had undetectable concentrations 80% of those who acquired HIV while on PrEP had undetectable TFV-DP None of the participants who acquired HIV had adequate adherence (≥ 4 doses/week)
**Global**											
Landovitz et al.^22^	Mixed populations across 72 PrEP studies	TDF 300 mg or FTC 200 mg	± 9¶	Adequate	≥ 700	4	§	§	§	§	HIV incidence rate 3.9 per 100 PY at < 2 doses/week (accounting for 83.3% of seroconversions) Incidence declined to 0.27 (4–6 doses/week) and 0.054 per 100 PY (≥ 7 doses/week).

Note: Please see full reference list of Alghamdi MK, Ali MW, Schoonees A, Kredo T, Decloedt E, Van Rensburg R. Adherence thresholds of tenofovir diphosphate in dried blood spots for HIV treatment and pre-exposure prophylaxis: A scoping review. S Afr J HIV Med. 2026;27(1), a1829. https://doi.org/10.4102/sajhivmed.v27i1.1829

DBS, dried blood spots; FTC, emtricitabine; IQR, interquartile range; MSM, men who have sex with men; NR, not reported; PY, person-years; PrEP, pre-exposure prophylaxis; s.d., standard deviation; TDF, tenofovir disoproxil fumarate; TFV-DP, tenofovir diphosphate.

† , Taking the number of prescribed doses needed to prevent HIV acquisition;

‡ , Taking all prescribed doses;

¶ , median follow up;

§ , not reported.

Hoenigl et al.^19^ defined adequate adherence as TFV-DP ≥ 719 fmol/punch (≥ 4 doses/week), a threshold derived from the reference study by Grant et al.^14^ The observed median TFV-DP concentration was 993 fmol/punch at four doses/week. However, the interquartile range (IQR) varied widely (0–1397 fmol/punch), indicating variability in individual adherence or TFV-DP PK.

Pyra et al.^20^ used the reference adherence thresholds of ≥ 700 fmol/punch (≥ 4 doses/week) for adequate adherence, as defined by Castillo Mancilla et al.^5^ and the extrapolated concentration of ≥ 1050 fmol/punch (≥ 6 doses/week) for optimal adherence, as defined by Anderson et al.^16^ Observed mean TFV-DP concentrations were 925 fmol/punch (standard deviation [s.d.]: 509) (4 doses/week), and 994 fmol/punch (s.d.: 517) (6 doses/week), which demonstrates moderate variability across participants. Among 150 DBS samples from the 103 participants, 87 samples (58%) met the lower adherence cut-off of ≥ 4 doses/week, and 62 (41%) met the ≥ 6 doses/week target.

Wahome et al.^21^ applied the reference adherence threshold of TFV-DP ≥ 700 fmol/punch (≥ 4 doses/week) for adequate adherence, based on Grant et al.’s study,^14^ However, the observed TFV-DP concentrations were notably lower than expected, with only 14.5% of participants reaching the adherence threshold. Among participants who acquired HIV while on PrEP, 80% had undetectable TFV-DP concentrations, suggesting poor adherence rather than PrEP failure. The HIV incidence was 3.6 per 100 person-years (PY) in MSM reporting PrEP use, and 5.4 per 100 PY in a concurrently followed internal comparator group who never started PrEP, indicating that suboptimal adherence may have contributed to the lack of risk reduction in this cohort.

In the large pooled analysis of 72 PrEP studies, Landovitz et al.^22^ evaluated TFV-DP concentrations in DBS as categorical adherence corresponding to dose equivalents. The majority of HIV seroconversions (83.3%) occurred in participants with TFV-DP concentrations consistent with < 2 doses/week, while HIV incidence declined substantially among those with higher adherence, reaching 0.27 per 100 PY in the 4–6 doses/week category, and 0.054 per 100 PY among participants with TFV-DP concentrations consistent with ≥ 7 doses/week.

#### Treatment studies

The eight treatment studies assessed the relationship between TFV-DP concentrations in DBS and virological suppression in PLWH receiving TFV-based ART (Table 4). Across multiple studies, higher TFV-DP concentrations were consistently associated with sustained virological suppression. For example, Castillo-Mancilla et al.^25^ applied TFV-DP > 1560 fmol/punch as the optimal adherence cut-off (i.e. taking all doses in a week) based on two reference studies.^5,14^ For 100% adherence, they reported observed median TFV-DP concentrations in DBS of 1847 fmol/punch (range 706–3776), and a median of 926 fmol/punch (range 706–1133) as adequate adherence in women who largely maintained HIV RNA < 20 copies/mL (*n* = 8/35), indicating virological suppression despite suboptimal dosing. Castillo-Mancilla et al.^9^ established a strong correlation between TFV-DP concentrations and virological suppression, with a threshold of ≥ 1850 fmol/punch, leading to a 73.5-fold increased likelihood of maintaining HIV RNA < 20 copies/mL versus < 350 fmol/punch. Similarly, Castillo-Mancilla et al.^27^ further demonstrated that only 21% of virologically suppressed participants (< 20 copies/mL) had TFV-DP ≥ 1850 fmol/punch and detectable emtricitabine triphosphate concentrations, indicating that some PLWH may maintain suppression despite suboptimal adherence. Moreover, Morrow et al.^26^ found that individuals with TFV-DP < 800 fmol/punch had a significant increase in the risk of developing viraemia. Specifically, compared with TFV-DP ≥ 1650 fmol/punch, TFV-DP < 800 fmol/punch was associated with 4.7-fold higher odds of future viraemia ≥ 20 copies/mL.

**TABLE 4:** Results of treatment studies from the United States and Africa.

Study Author	Participants	Intervention (daily)†	Duration	Objective	Threshold value for TFV-DP in DBS		Observed value for TFV-DP in DBS					Outcome
					Adherence	fmol/punch	Median (fmol/punch)	IQR (fmol/punch)	Mean for TDF-based regimen (fmol/punch)	Mean for TAF-based regimen (fmol/punch)	s.d.	
**United States**												
Castillo-Mancilla et al.^25^	Women with HIV	TDF 300 mg-based ART (TDF/FTC/ATV/r or TDF/FTC/RAL)	6 months	ART adherence	Optimal‡	1560	1874	706–3776	-	-	-	23% had TFV-DP < 1250 fmol/punch; 91% were virologically suppressed.
					Adequate§	< 1250	926	706–3776	-	-	-	
Castillo-Mancilla et al.^9^¶	Persons living with HIV	TDF 300 mg-based ART	12 months	Virological suppression (< 20 copies/mL)	Optimal‡	≥ 1850	‡‡	‡‡	‡‡	‡‡	‡‡	43% had TFV-DP ≥ 1850 fmol/punch. The aOR for virological suppression at this level was 73.5 vs. < 350 fmol/punch.
Castillo-Mancilla et al.^27^¶	Persons living with HIV	TDF 300 mg-based ART	3 months	Virological suppression (< 20 copies/mL)	Optimal‡	≥ 1850	2601	2199–3054	-	-	-	21% had TFV-DP ≥ 1850 fmol/punch; all had virological suppression despite some lower concentrations.
					Adequate§	NR	1658	1206–2295	-	-	-	
Morrow et al.^26^¶	Persons living with HIV	TDF 300 mg-based ART (TDF + FTC with NNRTIs, integrase inhibitors, or boosted PIs)	48 weeks	Future viraemia (≥ 20 copies/mL)	Optimal‡	≥ 1650	1704	BLQ–7432	-	-	-	The aOR of future viraemia: 4.7 (95% CI: 2.6–8.7) for TFV-DP < 800 vs. ≥ 1650 fmol/punch.
					Adequate§	≥ 800	1704	BLQ–7432	-	-	-	
Robbins et al.^24^	Young adults with perinatally acquired HIV	TDF 300 mg-based ART or TAF 25 mg-based ART	Ongoing cohort, adherence data collected ± 2 weeks	Future viraemia (> 200 copies/mL)	‡‡	‡‡	-	-	825	1847	826	Lower TFV-DP values associated with detectable VL TDF-based regimen: Mean 445.95 fmol/punch TAF-based regimen: Mean 309.11 fmol/punch.
**Africa**												
Jennings et al.^23^	Persons living with HIV	TDF 300 mg-based ART (TDF/FTC/EFV)	12 months	Future viraemia (> 400 copies/mL)	Adequate§	> 800	1041	727–1355	-	-	-	66.7% of viraemia cases had TFV-DP < 400 fmol/punch: OR 16.1 (95% CI: 3.9–67.4, *P* < 0.001) for future viraemia vs. > 800 fmol/punch.
Odayar et al.^7^	Postpartum women with HIV	TDF 300 mg-based ART (TDF/3TC/EFV or TDF/FTC/EFV)	24 months	Future viraemia (≥ 20 copies/mL)	Optimal‡	≥ 1850	1051	397–1466	-	-	-	25% had viraemia with TFV-DP ≥ 700 fmol/punch; 17% with ≥ 1850 fmol/punch. OR for < 350 vs. ≥ 1850 fmol/punch was 12.9.
Van Heerden et al.^28^	Persons living with HIV	TDF 300 mg-based ART (TDF/3TC /DTG)	48 weeks	Virological suppression (< 50 copies/mL)	Optimal‡	≥ 1250	1371	975–1958	-	-	-	Higher TFV-DP concentrations were associated with virological suppression (aOR: 4.69 at week 48) and lower risk of virological rebound.
					Adequate§	> 700	1371	975–1958	-	-	-	

Note: Please see full reference list of Alghamdi MK, Ali MW, Schoonees A, Kredo T, Decloedt E, Van Rensburg R. Adherence thresholds of tenofovir diphosphate in dried blood spots for HIV treatment and pre-exposure prophylaxis: A scoping review. S Afr J HIV Med. 2026;27(1), a1829. https://doi.org/10.4102/sajhivmed.v27i1.1829.

aOR, adjusted odds ratio; ART, antiretroviral therapy; ATV/r, atazanavir/ritonavir; BLQ, below the limit of quantification; CI, confidence interval; DBS, dried blood spots; DTG, dolutegravir; EFV, efavirenz; FTC, emtricitabine; FTC-TP, emtricitabine triphosphate; IQR, interquartile range; NNRTIs, non-nucleoside reverse transcriptase inhibitors; NR, not reported; PIs, protease inhibitors; PrEP, pre-exposure prophylaxis; s.d., standard deviation; TAF, tenofovir alafenamide; TDF, tenofovir disoproxil fumarate; TFV-DP, tenofovir diphosphate; RAL, raltegravir; 3TC, lamivudine; VL, viral load.

† , Doses of non-tenofovir ART drugs were not reported in the included studies;

‡ , Taking all prescribed doses;

§ , Taking the number of prescribed doses needed to achieve the outcome;

¶ , The Castillo-Mancilla et al.,^9^ Castillo-Mancilla et al.,^27^ and Morrow et al.^26^ studies were derived from overlapping or related cohorts, and should not be interpreted as fully independent datasets;

‡‡ , not reported.

In the South African setting, Jennings et al.^23^ reported that TFV-DP concentrations < 400 fmol/punch were strongly associated with future viraemia (> 400 copies/mL; odds ratio [OR]: 16.1, 95% confidence interval [CI]: 3.9–67.4). Odayar et al.^7^ studied postpartum women and found that 25% of viraemic events occurred even with TFV-DP ≥ 700 fmol/punch (vs 17% with ≥ 1850 fmol/punch). Importantly, women with TFV-DP < 350 fmol/punch had 12.9-fold higher odds of viraemia (≥ 20 copies/mL) compared to those with ≥ 1850 fmol/punch. Van Heerden et al.^28^ evaluated TFV-DP concentrations in DBS among adults initiating tenofovir-lamivudine-dolutegravir (TLD) as first- or second-line ART in South Africa. Higher TFV-DP concentrations were associated with virological suppression and lower risk of virological rebound, with TFV-DP concentrations assessed as a continuous variable performing better than categorical thresholds for predicting virological outcomes.

In addition, Robbins et al.^24^ showed that TFV-DP concentrations in DBS were inversely correlated with HIV viral load, with lower concentrations associated with viraemia (> 200 copies/mL) and higher concentrations linked to virological suppression (< 20 copies/mL). These corresponded with mean TFV-DP concentrations of 825 fmol/punch versus 446 fmol/punch, respectively, for tenofovir disoproxil fumarate (TDF) regimens, and 1847 fmol/punch versus 309 fmol/punch, respectively, for tenofovir alafenamide (TAF) regimens.

In the treatment studies, a TFV-DP concentration of ≥ 1850 fmol/punch was reported as a threshold associated with virological suppression in three out of eight studies.^7,9,27^ Other reported benchmarks included a TFV-DP concentration of 1560 fmol/punch as the target for optimal (100%) adherence,^25^ < 800 fmol/punch as a predictive threshold for future viraemia,^26^ and TFV-DP ≤ 400 fmol/punch as a strong predictor of viral breakthrough.^23^ Although the cut-off points varied across studies, these differences reflect distinct adherence categories and virological outcomes. Collectively, the findings consistently demonstrate that higher TFV-DP concentrations in DBS are associated with sustained virological suppression. Figure 2 summarises the reported TFV-DP thresholds across reference, PrEP, and treatment studies.

**FIGURE 2:**
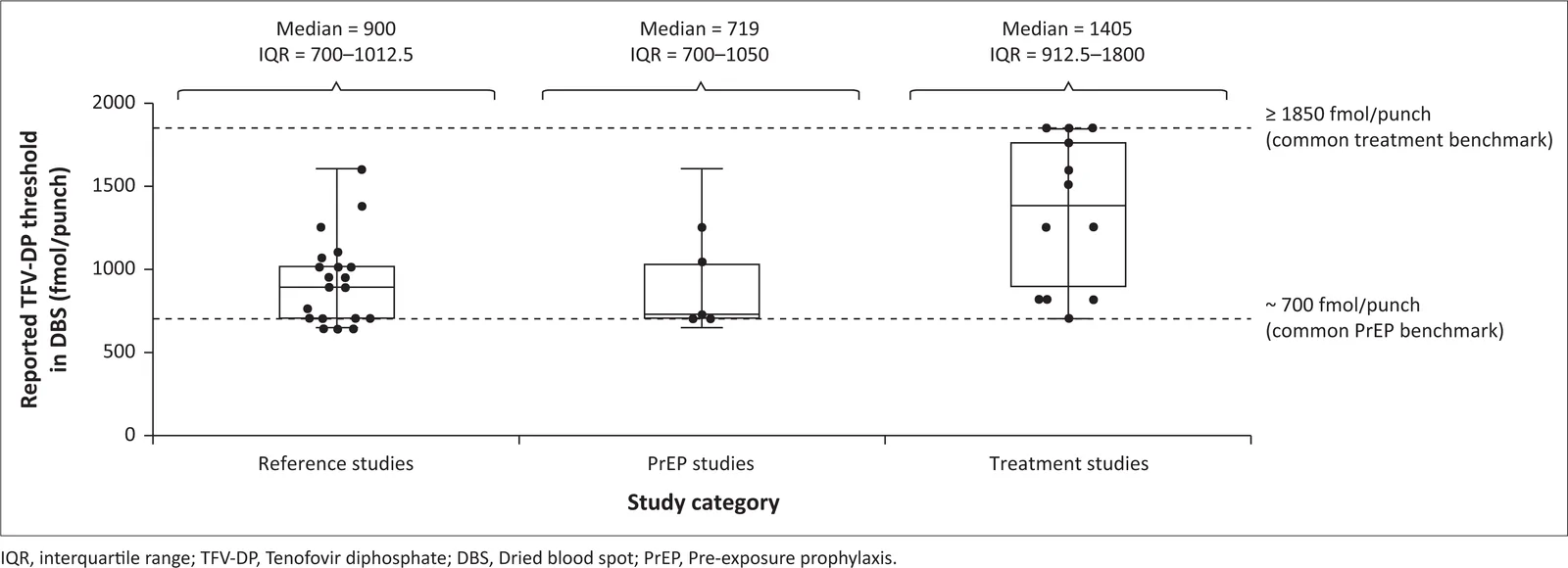
Tenofovir diphosphate thresholds across study categories.

Individual dots represent TFV-DP threshold values reported in included studies. Vertical lines represent the range of reported thresholds within each study category. Boxplots represent descriptive summaries (median and interquartile range) of the reported TFV-DP threshold values plotted within each study category. The dashed horizontal lines indicate the commonly reported benchmark for each category. Thresholds represent adherence-, HIV prevention-, virological suppression-, or future viraemia-associated TFV-DP concentrations according to the primary objective of each study.

## Discussion

This scoping review provides a comprehensive synthesis of the relationship between TFV-DP concentrations in DBS, adherence thresholds, and clinical outcomes for both PrEP and HIV treatment. Reported thresholds ranged from ≥ 650 fmol/punch for adequate adherence, to ≥ 1850 fmol/punch for optimal adherence, depending on the purpose of ART use, study population, and outcome.

The variability in threshold values reflects differences in the required TFV drug exposure for various clinical objectives – namely, HIV prevention versus treatment – as well as underlying biological and methodological factors. For instance, PrEP studies^19,20,21,23,24,25^ often used 700 fmol/punch as a conservative threshold for protection, while treatment studies^7,9,23,24,25,27^ linked higher concentrations (≥ 1250 fmol/punch – 1850 fmol/punch) to virological suppression. Differences observed in certain populations, such as lower concentrations during pregnancy or higher concentrations in individuals with lower body weight, suggest that fixed adherence cut-offs may not apply universally, and should be interpreted in context.

Several pharmacological, physiological, and methodological factors contribute to inter-individual and inter-study variability in TFV-DP concentrations in DBS.^29^ Despite this predictable kinetic profile, substantial variability is observed between individuals with similar dosing histories. ART regimen may influence TFV-DP concentrations independent of adherence, with higher concentrations observed with TAF-based regimens,^24^ and lower concentrations with efavirenz-based regimens (unpublished data), which should be considered when interpreting DBS-based thresholds. In addition, dolutegravir-based regimens exhibit greater forgiveness, with virological suppression often maintained despite missed doses, reducing the strength of the near-perfect adherence-to-viral load associations required with older regimens.^30^ Consequently, virological failure may occur less frequently at lower TFV-DP concentrations in this setting.^30^ These findings suggest that TFV-DP thresholds derived from efavirenz-based cohorts may not be directly generalisable to dolutegravir-based ART, particularly in African treatment programmes where dolutegravir-based regimens now predominate. While some studies identified in this scoping review evaluated the combined relationship between TFV-DP concentrations, adherence, and virological failure in ART recipients, limited data currently exist evaluating TFV-DP thresholds, specifically, in dolutegravir-based ART populations. However, the South African study by Van Heerden et al.^28^ demonstrated that TFV-DP concentrations analysed as absolute values rather than conventional categorical thresholds better predicted virological outcomes in individuals initiating TLD, arguing that dichotomised thresholds may be too restrictive when used to predict clinical outcomes with TLD.

Physiological factors may also play a role in variability, where individuals with lower body weight, or women, have been shown to exhibit higher TFV-DP concentrations.^31^ In contrast, conditions that reduce red blood cell lifespan, such as pregnancy, anaemia, or haemolytic disorders, may lead to lower red blood cell TFV-DP concentrations in DBS, despite adequate adherence.^8,31^ During pregnancy, TFV-DP concentrations can be up to one-third lower than postpartum concentrations, likely as a result of increased plasma volume, renal clearance, and changes in haematological parameters.^8^ Consistent with this, PK studies in peripartum women receiving daily PrEP under observed dosing conditions have demonstrated lower TFV-DP concentrations during pregnancy compared with postpartum, although no thresholds were advised.^32^ Clinical factors add further complexity: TFV-DP values can be influenced by haematocrit outside the 35% – 50% range, the use of finger-stick versus venipuncture (with capillary blood yielding ~ 10% lower values), and pre-steady-state sampling, which may require mathematical correction.^33^

Analytical variability further contributes to the observed heterogeneity in TFV-DP concentrations. Factors such as DBS punch size and placement, filter paper characteristics, storage conditions, extraction protocols, and laboratory assay calibration have all been shown to influence TFV-DP quantification: for example, switching from a 70:30 to a 50:50 methanol–water extraction mixture improves TFV-DP recovery from the DBS punch.^5,33^ This was demonstrated in the recent work by Mugwanya et al., that showed that extraction-method-specific conversion factors can materially shift TFV-DP concentrations, underscoring the importance of aligning adherence interpretation with laboratory methodology.^18^ Differences in assay requirements are also formulation-dependent: for example, accurate quantification of TFV-DP with TAF-based regimens often requires larger bloodspot volumes compared to TDF-based regimens, complicating direct comparisons.^10^ Overall, inter-assay variability has been estimated at approximately 18%, underlining the need for standardisation across laboratories.^15^ These factors highlight the need to consider the clinical and methodological context when interpreting TFV-DP concentrations.

Key gaps include the uncertainty of validated thresholds across diverse populations in different settings and regions, and limited data for subgroups such as pregnant women, adolescents, and older children who are eligible for TFV-based ART. Notably, lower adherence rates were also reported in studies conducted in low- and middle-income countries, which may be because of structural, economic, or health system challenges that influence medication-taking behaviour.^21,33^ While we did not establish universal thresholds, this review contributes to the body of knowledge by mapping existing values, contextualising their use, and highlighting the need for population-specific validation and methodological harmonisation in future research. Emerging evidence from African women challenges earlier assumptions that substantially higher adherence is required for PrEP efficacy in women compared with men.^18^

### Limitations

Our review has several limitations. As this was a scoping review, we did not formally appraise study quality; findings provide a descriptive overview rather than definitive TFV-DP thresholds. The heterogeneity of study designs, populations, and assays complicate direct comparisons of TFV-DP thresholds. Additionally, some included studies represented different analyses of overlapping or related cohorts, particularly the studies by Castillo-Mancilla et al.^9,27^ and the Morrow et al. study.^26^ As a result, certain thresholds may have been represented more frequently in the literature despite being derived from related participant populations, potentially amplifying the apparent consistency of specific TFV-DP benchmarks. Nevertheless, these studies addressed different research questions and evaluated different clinical outcomes. Most studies were conducted in high-income countries, limiting generalisability to low-resource environments where adherence may be affected by limited access to healthcare, medication stockouts, transportation issues, stigma, and food insecurity. Women remain underrepresented in many PrEP studies, which have mainly enrolled MSM. Similarly, there is a lack of robust data on TFV-DP concentrations in DBS in children and adolescents, who may differ in drug exposure, adherence patterns, and clinical response. Despite these limitations, we attempted to minimise bias by conducting a comprehensive and unrestricted search to identify the available data. To account for clinical diversity, we categorised studies by indication (PrEP vs treatment) and interpreted TFV-DP thresholds within their respective clinical contexts rather than applying a universal standard.

### Future research directions

Future studies should confirm the relationship between TFV-DP concentrations in DBS, adherence patterns, and clinical outcomes in larger studies to enable translation of the cut-off values that are mostly used in research settings to clinical practice. These studies should also investigate underrepresented groups more extensively, such as pregnant women, adolescents, and children. Furthermore, because of the high cost currently associated with quantifying TFV-DP in DBS, innovative laboratory techniques should aim to develop more cost-effective methods to reduce costs and enable more widespread use, especially in low-income countries where the HIV burden is the highest and where most of the concentration variability was found. In addition, future work should prioritise harmonisation of DBS extraction methods and reporting standards to improve comparability across studies. Future studies should specifically evaluate TFV-DP adherence thresholds across different partner antiretroviral agents, particularly integrase inhibitor-based regimens such as dolutegravir.

### Clinical implication

By providing an objective, cumulative measure of adherence, TFV-DP concentrations in DBS can help identify individuals at risk of treatment failure, differentiate between nonadherence and viral evolution, and guide targeted adherence interventions to enhance the global HIV treatment and prevention efforts. While ~ 700 fmol/punch is a consistent PK-derived benchmark for PrEP corresponding to adequate adherence (approximately ≥ 4 doses/week), higher thresholds ≥ 1850 fmol/punch have mainly been associated with robust virological suppression in treatment studies, highlighting the different exposure-response dynamics with HIV prevention compared to treatment. However, these cut-offs should be applied cautiously, considering physiological variation, assay differences, and population-specific factors, until more robust evidence is available to translate to clinical practice.

## Conclusion

Despite the growing use of TFV-DP on DBS for HIV PrEP and treatment, a unified threshold that defines adequate adherence associated with beneficial clinical outcomes has not yet been identified. Instead, this review highlights the need for purpose- and population-specific thresholds given the notable variability in concentrations between populations and by PrEP or treatment indication. By integrating findings from clinical trials and PK modelling studies, this review provides a comprehensive framework for mapping population-specific adherence cut-offs. Our findings highlight the need for further investigation and the eventual confirmation of TFV-DP adherence thresholds to optimise both HIV treatment and PrEP strategies.
